# Selective Integrin α_5_β_1_ Targeting through Spatially Constrained Multivalent DNA-Based Nanoparticles

**DOI:** 10.3390/molecules27154968

**Published:** 2022-08-04

**Authors:** Eva E. Kurisinkal, Vincenzo Caroprese, Marianna M. Koga, Diana Morzy, Maartje M. C. Bastings

**Affiliations:** 1 Programmable Biomaterials Laboratory, Institute of Materials, School of Engineering, Ecole Polytechnique Fédérale Lausanne, 1015 Lausanne, Switzerland; 2 Interfaculty Bioengineering Institute, School of Engineering, Ecole Polytechnique Fédérale Lausanne, 1015 Lausanne, Switzerland

**Keywords:** selective targeting, spatial tolerance, DNA nanotechnology, integrins, multivalency

## Abstract

Targeting cells specifically based on receptor expression levels remains an area of active research to date. Selective binding of receptors cannot be achieved by increasing the individual binding strength, as this does not account for differing distributions of receptor density across healthy and diseased cells. Engaging receptors above a threshold concentration would be desirable in devising selective diagnostics. Integrins are prime target candidates as they are readily available on the cell surface and have been reported to be overexpressed in diseases. Insights into their spatial organization would therefore be advantageous to design selective targeting agents. Here, we investigated the effect of activation method on integrin α_5_β_1_ clustering by immunofluorescence and modeled the global neighbor distances with input from an immuno-staining assay and image processing of microscopy images. This data was used to engineer spatially-controlled DNA-scaffolded bivalent ligands, which we used to compare trends in spatial-selective binding observed across HUVEC, CHO and HeLa in resting versus activated conditions in confocal microscopy images. For HUVEC and CHO, the data demonstrated an improved selectivity and localisation of binding for smaller spacings ~7 nm and ~24 nm, in good agreement with the model. A deviation from the mode predictions for HeLa was observed, indicative of a clustered, instead of homogeneous, integrin organization. Our findings demonstrate how low-technology imaging methods can guide the design of spatially controlled ligands to selectively differentiate between cell type and integrin activation state.

## 1. Introduction

A significant hurdle in the administration of therapies, and, in particular, in combatting infections, cancers and genetic diseases, is in the specific delivery of the therapeutic agent to targeted elements of an organ, tissue or cell type, i.e., active targeting [1]. Cell receptors are often employed as targeted elements, due to varying expressions dependent on cell/tissue type and accessibility for binding [2]. Prime candidates amongst cell receptors are integrin transmembrane receptors, reportedly differently regulated in diseases [3,4]. Integrins are bi-directional signaling receptors between the cell and its extracellular environment, also mediating migration, proliferation, and differentiation. The 18α and 8β integrin subunits associate forming 24 heterodimers that cluster with functional and tissue specificity [5]. The expression of integrins, whilst ubiquitous, is cell-type dependent with respect to the individual heterodimers and account for heterodimer function [6,7]. As such, integrins are spatially regulated and have the capacity to respond to changes in the extracellular environment and transduce such changes to the intracellular environment [8,9]. These differences in expression make integrins an ideal candidate to engineer materials that allow for super-selective targeting, [10] e.g., using the density of receptors as a switch to define the onset of binding.

The integrin α_5_β_1_ has been implicated in several diseases, ranging from cancer, inflammation [11], respiratory diseases [12], neurological disorders [13] and viruses, including SARS-CoV-2 [14], besides being engaged in cell adhesion [15]. Upregulation of integrin α_5_β_1_ expression in epithelia is associated with inflammation [16] and active proliferation [17]. Changes in the polarization of epithelial integrin expression is reported in transformation from normal to malignant states [18]. Integrin α_5_β_1_ expression on the cell surface, versus relocation to the cell interior, of fibroblasts have also been implicated in resisting cancer progression [19] and has been proposed as a target for anti-angiogenic therapies, due to its role in tumor angiogenesis [20]. Altogether, the selective engagement of the integrin α_5_β_1_ subtype could lead to applications spanning the improvement in cellular uptake of drugs [21] and genetic material [22], the modulation of stem cell differentiation [23] and cell adhesion applications, e.g., artificial extracellular matrix (ECM) [24].

Integrins were first demonstrated to regulate their ligand-binding activity through conformational changes [25]. The conformational states adopted would, in turn, influence the availability of binding sites for integrin targeting and the spatial distribution of receptors within clusters. Applying super-resolution imaging techniques, integrin α_5_β_1_ on the cell surface has been reported to segregate into discreet focal adhesion clusters of active and inactive integrins, with active clusters having a higher local order [26]. When not engaged in adhesion cluster formation, a random surface distribution can be assumed. Integrin α_5_β_1_ was also shown to have nanoscale ligand spatial preferences distinct from other integrin subtypes with different ligand binding behaviors in the range of spacings below 60 nm [27]. The overall conformational changes, i.e., closed-bent at ~90° at rest to open-extended, observed in the integrin α_5_β_1_ expressed ubiquitously across cell types, are attributed to their primary function in cell adhesion [28]. Therefore, we hypothesize that the distribution of integrin subtypes is regulated by cell function, which motivates the analysis of spatially controlled targeting across cell types.

Given the different expression levels of subtypes of integrin receptors, based on cell type and state, engineering a targeting system capable of differentiating between these situations would have the potential to engage selective cell responses. From the pioneering works of Seeman [29,30] followed by the DNA origami folding method introduced by Rothemund [31], DNA nanotechnology has been employed in different cellular contexts, capitalizing on its exclusive spatial addressability and uniformity by design [32,33,34]. Using DNA-based scaffolds for the nanoscale manipulation of ligand presentation for cell receptor binding [34] is an ideal approach, due to a number of factors: (i) sequence programmability that imparts modularity in DNA architectures and ligand presentation, (ii) biocompatibility, (iii) facile tailoring of scaffold flexibility, (iv) mono-dispersity (v) high spatial precision, down to the Ångström level, and (vi) relatively low cost for synthesis. Multivalent nanoscale DNA scaffolds, therefore, provide an excellent platform to investigate spatially-controlled binding in an attempt to selectively engage clusters of integrins present on different cell types and states.

In this study, our aims are threefold. First, we focus on obtaining quantitative information of receptor spacing on surfaces of different cell types, and in different activation states, using readily available laboratory techniques and standard tools. The latter is important to generate a workflow that is generally applicable to any cell surface target, without the need for specialized equipment. Secondly, we hypothesize that receptor distributions are affected by activation state and that this metric can be a critical parameter in the design of selective targeting. We, thus, focus on the analysis of various activation methods and the quantitative effect of activation on receptor spacing. Finally, translating the spatial information of receptor distribution into DNA-scaffolded particles, we aim to demonstrate that by using spatially-controlled ligands, a selective interaction within cells, between activation states and between cell types can be obtained. This selective interaction can be observed already with bivalent ligands, if the scaffold spacing is chosen accurately.

## 2. Results

### 2.1. Analysis of Cell Type Specific Integrin α_5_β_1_ Expression Levels

#### 2.1.1. Choice of Cell Types

Integrin α_5_β_1_ is implicated in cell proliferation and angiogenesis essential for endothelial cell function [35] and demonstrates a loss in polarization upon epithelial malignant transformation [18]. An overexpression of integrin α_5_β_1_ in cervical cancer has previously been correlated with negative chemotherapeutic response and recurrence [36]. Additionally, epithelial upregulation of α_5_β_1_ expression is associated with inflammatory response [6] and active proliferation [7]. Based hereon, expression levels can be expected to differ significantly amongst these cell types. Therefore, we decided to investigate the relative integrin α_5_β_1_ expression levels in HUVEC (endothelial), CHO (epithelial) and HeLa (epithelial carcinoma) cells for studies probing inter-receptor spacing and distribution.

#### 2.1.2. Activation of α_5_β_1_

**Figure 1:**
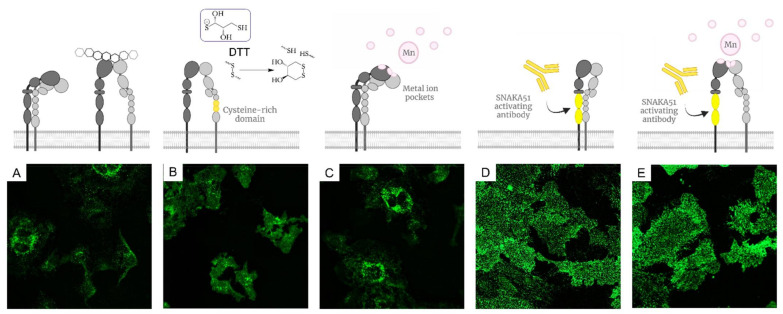
Methods for integrin α_5_β_1_ activation. Fluorescence microscopy images (**A**–**E**) show the resulting staining of fluorescently labeled SNAKA51 on HUVECs (**A**) in resting conditions (**B**) activated with DTT (**C**) in presence of Mn^2+^ (**D**) following initial SNAKA51 incubation (**E**) in combination of SNAKA51 and Mn^2+^.

Integrin conformation, clustering and spatial distribution is initiated and influenced by the integrin activation method employed [37,38]. We conducted this analysis to determine the activation method best suited for use in our future studies. First, we analysed the effectiveness of different activation methods, notably divalent cation Mn^2+^ [39], an activation-inducing antibody SNAKA51 [40] and reducing agent dithiothreitol (DTT) [41], on integrin cluster distribution. HUVECs have high expression levels of integrin α_5_β_1_ as they are key modulators of endothelial cell function in regulating cell engagement with the ECM. Interestingly, the difference in the effect of the respective activation conditions on both HUVEC morphology and integrin α_5_β_1_ distribution was pronounced (Figure 1). The observed fluorescence localisation was in good agreement with previous reports on the SNAKA51 labeling of integrin α_5_β_1_ at the farther edge of focal adhesions, distal from the cell perimeter and at the sites of fibrillar adhesion [40]. The sole use of DTT and Mn^2+^ as activating condition was found insufficient in specifically driving α_5_β_1_ activation. Yet for Mn^2+^ in combination with SNAKA51, integrin α_5_β_1_ clusters and cell morphology in HUVEC were indistinguishable from SNAKA51 staining alone. As precipitation in DNA compatible buffers was observed with addition of Mn^2+^, we decided to employ solely the SNAKA51 antibody as an activating method. Importantly, the SNAKA51 binding epitope is specific to the integrin α_5_β_1_ and is situated far from the ligand binding site, unlikely to sterically hinder the binding of subsequent liganded DNA nanoparticles.

#### 2.1.3. Model of Integrin α_5_β_1_ Distributions

**Figure 2:**
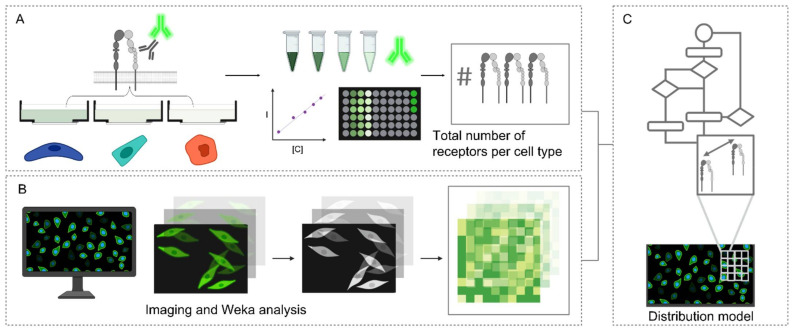
Workflow in modeling integrin α_5_β_1_ receptor distributions in HUVEC, CHO and HeLa. (**A**) Immuno-staining assay and subsequent comparison with a calibration curve to calculate total number of receptors (**B**) Fluorescence microscopy and image processing by Weka analysis (**C**) Mathematical model of integrin α_5_β_1_ distribution of interreceptor spacings.

Obtaining information on receptor distances for an informed materials design of ligand spacings is critical in spatial-controlled targeting. To date, crystallography data [42,43], if present, or super-resolution imaging [44], have been used to analyze inter-receptor distances. Often, receptors on the cell surface are mobile and a ballpark range of spacing suffices. Therefore, we wanted to establish an easy-to-implement strategy that did not require advanced super-resolution imaging techniques or expensive reagents. We set out to establish a method to interrogate the integrin α_5_β_1_ nearest neighbor spatial distributions in the three cell types displaying different integrin α_5_β_1_ overall expression levels. The activated HUVEC, CHO and HeLa cells were subjected to a workflow for the modeling of integrin α_5_β_1_ interreceptor distributions, detailed in Figure 2. First, an immuno-staining assay was performed on the HUVEC, CHO and HeLa and quantified with both fluorescence intensity measurements and imaged by fluorescence microscopy (Figure 2A). Fluorescence intensity measurements from the secondary antibodies in the respective conditions were derived from an established calibration curve. An assumption of 1:1 binding was employed in the ratio of secondary antibody to receptor binding.

**Figure 3:**
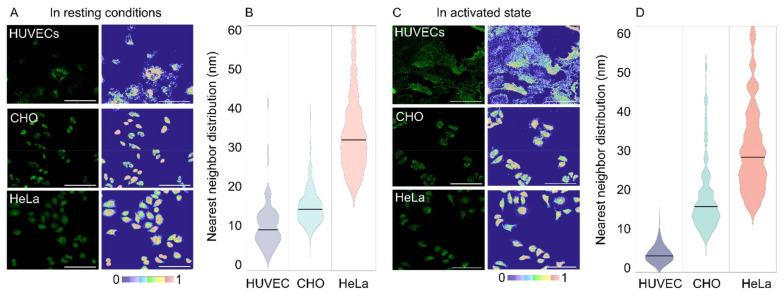
α_5_β_1_ expression and distribution in HUVEC, CHO, and HeLa cells. (**A**) Representative fluorescent microscopy images and their projected integrin density heatmap for resting conditions. (**B**) Global integrin nearest neighbor distributions across cell types in resting conditions. Horizontal lines represent the median values. (**C**,**D**) similar to (**A**,**B**) for integrins in their activated state. Scale bar = 100 µm.

For the modeling of receptor distributions, 3 images were selected and used for training with the Trainable Weka Segmentation FiJi plugin [45]. This generated classifier models distinguishing between cell pixels and background pixels for the respective conditions (e.g., cell type and activation state), which were used in the Weka analysis FiJi plugin developed in house, detailed in (Supplementary S1). The number of integrin α_5_β_1_ receptors were distributed by pixel intensity and the inter-receptor distances within a pixel (3.115 pixels µm^−1^) were calculated assuming an even distribution between pixel intensities, factoring a jamming limit of ~0.55 (Figure 2B,C) [46,47]. This step could cause an overestimation of distances, as local clusters that fall under the resolution limit would not be accounted for. Representative images of expression of the α_5_β_1_ integrin in each cell line with corresponding integrin heatmaps are visualized in Figure 3A,C. The normalized distributions of integrin α_5_β_1_ receptor spacings following our full workflow are plotted in Figure 3B,D. A detailed workflow can be found in (Supplementary S2), and reference values for colourmap generation in (Supplementary S3). The obtained data show clear differences in spatial integrin organization between activated and non-activated states in HUVECs, though less differences in distributions between resting and active state were noted for CHO and HeLa. CHO distributions seem more clustered in resting conditions and spread to larger spacings when activated. Between the three cell types, an increasing integrin spacing was observed from ~10 to ~20 to ~30 nm for HUVEC to CHO to HeLa, respectively. We expected these spacing differences to be sufficiently large to allow for spatially-controlled targeting with DNA scaffolds.

### 2.2. Design of a Spatially Constrained DNA Nanoparticle Library

**Figure 4:**
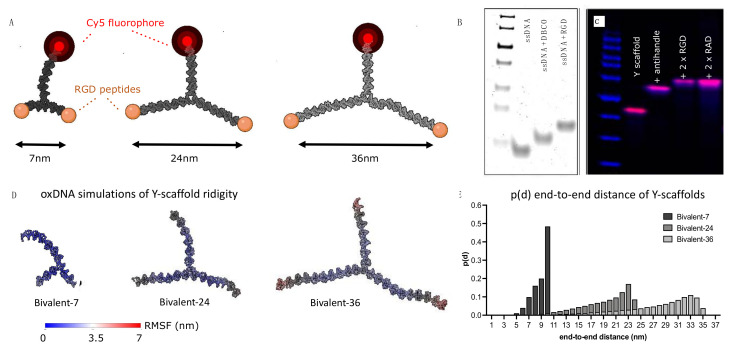
Bivalent DNA scaffold library with increasing ligand spacing. (**A**) Snapshots from simulated Y-scaffolds, with schematically added labels (red) for detection and RGD ligands (orange) for integrin binding. (**B**) PAGE gel analysis of DNA-peptide conjugation. (**C**) Agarose gel analysis of Y scaffold assembly; Lane 1: Y-scaffold; Lane 2: Y-scaffold + ssDNA anti-handles; Lane 3+4: Peptide-functionalized Y-scaffold (blue = DNA ladder, pink = Cy5 label). (**D**) OxDNA simulation of the Y-scaffold library, with representation of the root mean square fluctuation (RMSF). (**E**) Inter-ligand spacing probability distribution p(d) based on oxDNA simulations.

The modeled integrin nearest neighbor distance was translated into a ligand end-to-end distance of a Y-shaped DNA nanoparticle library (Figure 4A). The fibronectin based minimum cell adhesion sequence arginine-glycine-aspartate (RGD) [48,49] is known to interact with the active state of integrin receptors. The low spatial requirements of RGD peptides allow them to be packed with higher densities on the cell surface, compensating for relatively low monovalent adhesion affinity [50]. Two arms were used for RGD ligand presentation via classical ssDNA handle–anti-handle interaction (Figure 4B,C) and the resulting bivalent scaffolds were designed to have ligand spacings of approximately 7 nm, 24 nm and 36 nm (Supplementary S4). The ligand spacings attributed to the respective scaffolds were calculated and rounded up to the nearest nanometer calculated for a maximum arm-to-arm distance conformation of 180° between scaffold arms. Arm-to-arm spacings could be assumed rigid as double stranded DNA (dsDNA) is estimated to have a persistence length of ~150 base pairs or ~50 nm, which we were well below [51]. To further confirm the spacing of the ligands, we performed oxDNA simulations [52] (Figure 4D) and calculated the probability distribution p(d) of all particles (Figure 4E). While oxDNA overestimates the flexibility in the three-way junction design [53], the obtained results confirmed the predicted spacing, as well as predominant rigid behavior of our library. For the smallest scaffold, bridging between neighboring integrins into a tight cluster is expected, as this distance is situated in the range reported to be the diameter of the integrin headpiece (5–10 nm) [28,54]. The larger spacings are expected to interact with sparser integrin densities, as both RGD ligands need to interact in order to establish a robust interaction. The third arm of all scaffolds was kept constant and used for dye presentation to allow for particle detection (Supplementary S5 and S6).

### 2.3. Analysis of α_5_β_1_ Targeting Based on Spatial Selectivity

**Figure 5:**
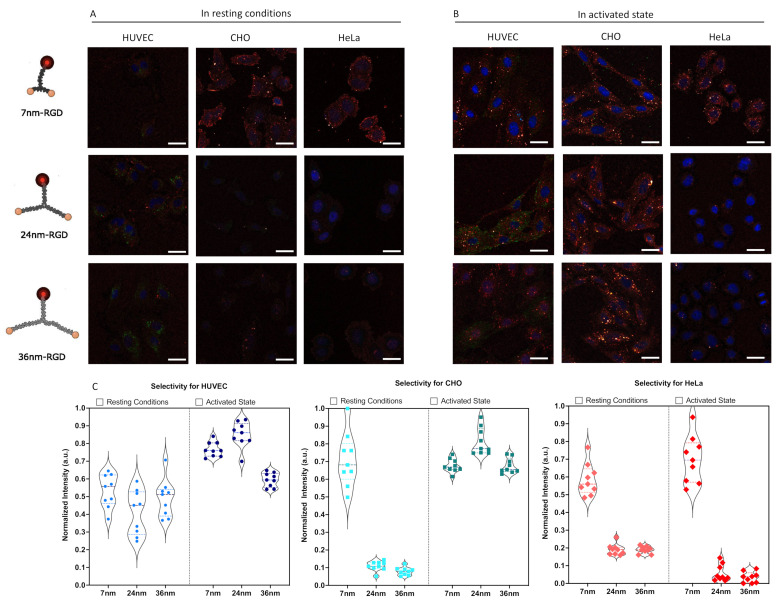
Confocal microscopy analysis of spatially constrained integrin staining. (**A**) Representative overview for non-activated cells (**B**) Representative overview for activated cells. Images show the DNA-analytes in red (Cy5 label), antibody-stained integrin in green (Alexa 488) and the nucleus in blue (DAPI). Scale bar = 30 µm (**C**) Quantification of selective binding, in resting conditions and in activated state. Plotted is the globally normalized intensity of scaffold present within the cell boundaries from a Z-projection of confocal images, corrected for background. Data is quantified from nine independent image stacks containing multiple cells per image. A full statistical analysis can be found in Supplementary S9.

Confocal microscopy images were obtained after incubation with the respective RGD modified DNA scaffolds on HUVEC, CHO and HeLa, in resting (Figure 5A) and activated (Figure 5B) cell conditions. To prevent false positive data from a monomeric ligand interaction, the effective concentration regime for bivalent binding was determined (Supplementary S7), which lay comfortably lower than the monovalent *K_d_*. RAD non-binding peptide ligands were included to confirm no a-specific interactions were present (Supplementary S8). The selectivity of each Y-scaffold per cell type was quantified by calculating the levels of Cy5 signal within the cell boundaries after a Z-stack projection through summation of slices. The signal in the nucleus was excluded. Only datasets that resulted in higher signal compared to the background were considered in our analysis (Figure 5C).

In resting conditions, no pronounced ligand selection was observed for HUVECs. CHO and HeLa cells. However, they had trends in efficacy favoring the 7 nm-RGD scaffold. Interestingly, the 7 nm-RGD-ligand interacted with CHO cells in resting conditions, predominantly staining the cell and nuclear periphery, regions where the integrin α_5_β_1_ is known to reside in nascent and fibrillar adhesions, respectively [55]. These observations matched the heatmap results from our model, where local high-density regions were found in CHO. Whilst the signal was not as marked on the cell periphery, small clusters were observed on the entirety of the HeLa cell surface with larger clusters in the nuclear periphery. The staining on CHO and HeLa by the 7 nm scaffold suggested the presence of densely clustered, neighboring integrins.

In activated state, HUVECs showed an increased preference for the 7 nm-RGD and 24 nm-RGD scaffolds, which followed, in part, the predictions from the spatial analysis model. From an overall increased density of integrins, one could indeed expect the favored selectivity of the smaller scaffolds. For CHO, similar to the model, a shift toward a larger spacing selectivity was observed in the experimental data. For both HUVEC and CHO, the selectivity in activated state was significantly different than in resting conditions (Supplementary S9). HeLa, however, continued to prefer the shortest ligand spacing, contrary to the largest spread that was measured in the theoretical model. Consistent with the model, however, was that, for HeLa, no significant changes in selectivity between resting and activated state were observed. The lack of difference between HeLa when activated versus in resting conditions of integrin α_5_β_1_ extended conformations, could be attributed to the upregulation of integrin α_5_β_1_ in certain solid tumors, including cervical cancer [56], forming unliganded clusters. These clusters of unliganded integrins are reportedly modulated by cancer-specific intracellular-binding factors promoting cancer progression through the sensing of ECM composition [57]. Altogether, we clearly observed that the same particle interacted significantly different between cell types and between activation states within the same cell type. Interestingly, this selectivity is already significant for a bivalent ligand presentation, as long as the scaffold is sufficiently rigid to ensure the required spatial-organization.

## 3. Discussion

The importance of understanding the biases in the spatial distribution of integrins is significant, both for our understanding of cellular function as well as of disease progression. The resulting information would advance targeted therapies through the intelligent design of materials capable of selective eliciting of cellular responses through the incorporation of multivalent interactions. Here, we aimed to engineer scaffolds which presented matching ligand to receptor spacing, capitalizing on the unique spatial tolerance inherent to DNA nanotechnology. DNA-scaffolded bivalent RGD ligands were engineered displaying a defined spatial organization, following the regimes of integrin α_5_β_1_ receptor spacing modeled through a custom workflow of image analysis of antibody-stained cell surfaces. Our experiments focused on demonstrating how an accessible receptor-spacing workflow could be used to design DNA guided ligand presentation and obtain selective binding on surfaces with different receptor densities across HUVEC, CHO and HeLa in resting and activated conditions.

In our novel imaging workflow and model, we demonstrated the possibility to extract global receptor spacings from low-technology immuno-stained cell experiments. This approach relied merely on the existence of specific antibodies to the target receptor. The results of the modeled distributions of α_5_β_1_ spacings suggested sufficiently diverse expression levels across the cell types, which provided a platform for investigating the modeled integrin α_5_β_1_ spacings with spatially constrained liganded DNA scaffolds. Of note, is that this technique is limited to the pixel size and minimal fluorophore detection limit. Within a pixel, an even distribution of receptors is assumed. However, given the dynamic nature of proteins on the cell surface, we believe this approximation is acceptable if global spacing information is required. Yet, when target receptors are known to, or suspected to, form nanoclusters, it is evident that the global neighbor distance from our method would present an overestimation of space. For the detection limit, since low signal equals low receptor concentration equals large spacing, these events would typically fall outside of a single nanoparticle detection distance, and, hence, would not affect the method. While the level of detail obtained through super-resolution methods cannot be matched, the facile accessibility and low cost of our method factor in the balance when the highest detail is not required.

A library of RGD liganded scaffolds was engineered to present the peptide ligands in a bivalent manner with spacings derived from the modelled α_5_β_1_ nearest neighbor distances. We showed that the efficacy of integrin α_5_β_1_ binding with these bivalent ligands strongly depended on the ligand spacing. An important factor is the boundary condition that measurements are performed under the monovalent ligand affinity concentration, to exclude monomeric contributions in binding and solely screen for the multivalency effect. Using the regime specific for bivalent α_5_β_1_ binding simultaneously reduces any contributions of RGD interactions with other integrins, though cannot completely rule them out. Of general note, is that a careful testing of the ligand’s specificity to its expected target receptor should be executed, as cross-reactivity would affect the spacing balance and negatively contribute to selective targeting.

The trends in binding efficacy observed across RGD scaffolds in function of α_5_β_1_ in extended conformation can be explained as a trade-off in terms of the following: (i) a match of inter-receptor spacing to ligand presentation, (ii) number and density of integrins within a cluster [10] (iii) local cluster topography due to thickness of glycocalyx [58] (iv) potential binding sites per surface area probed (v) entropic penalty vs. enthalpic gain in scaffold binding through relatively rigid ligand presentation [59,60]. To elaborate on the complexity in the interpretation of the binding trends observed in terms of trade-off between glycocalyx thickness and enthalpy-entropy compensation, a thicker glycocalyx increases integrin-mediated cell adhesion by entrapment in an activated state once extended from the cell surface [61]. From the perspective of an integrin-liganded scaffold, a thick glycocalyx would sterically hinder binding site accessibility, yet stabilize binding events once they occurred. As endothelial cells have a thicker glycocalyx [62] and cancer cells frequently overexpress components of the glycocalyx [63] the trends observed on activated HeLa cells with the larger 24 nm-RGD and 36 nm-RGD could be explained as a negative enthalpic-entropic compensation [64]. The 7 nm-RGD, which has a lower entropic penalty upon binding, due to a reduced multiplicity of conformations, lower loss of translational and rotational entropy related to molecular weight [59], and higher “effective molarity” [65], might further contribute to its gain in enthalpic–entropic compensation if “trapped” by the cells’ glycocalyx, and, hence, benefits from a favored overall binding. Overall, the integrin receptor, with its different conformations and subtypes present on the cell surface, presents a complex landscape to study interactions. Yet, even within this complexity, the overall power of spatially-controlled targeting using precisely engineered scaffolds was apparent.

Concluding, our work presents the cell selective α_5_β_1_ integrin targeting using bivalent, spatially controlled scaffolds designed by spatial input from a low-technology, easy-to-implement method to obtain quantitative information on global receptor spacing on the cell surface. We demonstrated the workflow using the α_5_β_1_ integrin, a critical regulator of cell-adhesion but equally important in many diseases. Indeed, a significant cell-type dependent spacing-signature profile was obtained, with clear differences when cells were activated. Translating the spatial information of receptor distribution into DNA-scaffolded multivalent particles, we demonstrated selective interaction within HUVEC and CHO cells, and between their activated and resting state. As the methodology homogenizes the receptor spacing within a pixel, deviations from reality in the special scenario when receptors form nanoclusters, can be expected. This was indeed observed when analyzing HeLa cells. Taken together, the accessibility of the presented approach, combined with the demonstration of differential targeting based on divalent ligand spacing, presents a broadly applicable strategy when selective cell targeting is envisioned.

## 4. Materials and Methods

Unless otherwise specified, reagents were used as received without further modification. Deionized water obtained from a Milli-Q water purification system was used for all experiments. Penicillin/streptomycin (Cat.No. 15140122), DMEM + GlutaMAX (Cat.No. 31966021), Fetal Bovine Serum (Cat.No. 10500064), DPBS (Cat.No. 14040091), 0.05% Trypsin-EDTA (Cat.No. 25300054), BlockAid blocking solution (Cat.No. B10710) and BSA 7.5% solution (Cat.No. 15260037) were purchased from Thermo Fisher Scientific, Waltham, MA, USA. All buffers were filtered with 0.22 µm PES syringe filters (Cat.No. 431229, Corning, NY, USA) or PES bottle filters (Cat.No. 431097, Corning). HeLa Ohio cells were obtained from SuNMIL Lab, EPFL and CHO from the EPFL Protein Facility, Lausanne, Switzerland. HUVEC (Cat.No. C2517AS), Reagent Pack™ Subculture Reagents (Cat.No. CC-5034) and EGM2 endothelial cell growth medium-2 bulletKit (Cat.No. CC-3162) was purchased from Lonza, Basel, Switzerland. 96 well ibidi angiogenesis µ-plates (Cat.No. 89646, Vitaris AG, Baar, Switzerland), Poly-L-lysine solution (Cat.No. P4832, Merck, NJ, USA). Rabbit anti-Integrin alpha 5 antibody EPR7854 (ab150361) 1:200, Recombinant Rabbit IgG, monoclonal (Cat.No. ab172730) 1:1500, Goat Anti-Rabbit IgG H&L Alexa Fluor^®^ 488 (Cat.No. ab150077) 1:500, and Goat Anti-Mouse IgG H&L Alexa Fluor^®^ 488 (Cat.No. ab150113) 1:500 were purchased from abcam, Cambridge, UK. Integrin alpha 5/CD49e Antibody (SNAKA51) (Cat.No. NBP2-50146) 1:1000 was purchased from Novus Biologicals, CO, USA. Mouse IgG2a kappa, clone eBM2a, eBioscience (Cat.No. 15287367) and 4%PFA (Cat.No. 15424389) were purchased from Fisher Scientific, NH, USA. Fmoc-Rink Amide MBHA resin (Cat.No. AS-20083, Anaspec, CA, USA), DBCO-maleimide, (Cat.No. 760668, Merck). All ssDNA strands listed below were ordered from Integrated DNA Technologies (IDT), Coralville, IA, USA.

### 4.1. Cell Activation and Antibody Binding Assay

HUVEC, CHO and HeLa cells were seeded in 96 well ibidi angiogenesis µ-plates, 3000 cells per well. Cells were incubated overnight at 37 °C, 5% CO_2_, 95% relative humidity. When relevant, cells were incubated in media supplemented with activator (DTT 3 mM, Mn^2+^ 1 mM, SNAK primary antibody 1 µg mL^−1^, or a combination of Mn^2+^ 1mM and SNAK primary antibody 1 µg mL^−1^) for 45 min. The following steps were performed at RT. Cells were fixed in 2% PFA for 15 min, washed with DPBS, and blocked for 1 h with BlockAid blocking solution. The cells were then incubated on a shaker for 1 h with the respective primary antibodies diluted in BlockAid. Cells were washed with DPBS supplemented with 3% BSA and incubated with the respective secondary antibodies diluted in BlockAid for 1 h.

For calibration, 96 well ibidi angiogenesis µ-plates were coated with poly-L-lysine (PLL) for 1 h and washed with DPBS prior to incubation with a serial dilution of the Alexa Fluor^®^ 488 conjugated secondary antibodies for calibration of antibody concentrations in experimental wells. Calibration wells were washed with DPBS prior to fluorescence measurements. Fluorescence intensities were measured on a BioTek™ Cytation 5™ Cell Imaging Multi-Mode Reader, BioTek Instruments, Inc. using the Gen5 software, Version 3.10. Monochromators were used to measure the fluorescence intensities of the Alexa Fluor^®^ 488 secondary antibodies (BioTek, Ex/Em 495(10)/519(10)). A 20X PL FL Phase (Olympus, LUCLFLN 20X) phase objective was used. Imaging settings were kept constant across cell lines when comparing different conditions. Dimensions 4X images: 1224 × 904 pixels (0.619 pixels µm^−1^) Dimensions 20X images: 1224 × 904 pixels (3.115 pixels µm^−1^).

### 4.2. Ligand Synthesis and DNA Conjugation

Peptide synthesis of RGD peptides with a GC linker was conducted, based on previously reported synthesis [66]. A 65 µmol Fmoc-based solid phase peptide synthesis (SPPS) was conducted of the following peptides (GRGDSGGGC, GRADSGGGC) on Fmoc-Rink Amide MBHA resin. Peptides were conjugated to ssDNA strands with azide modification. All the following steps were conducted at RT. SsDNA was resuspended in 0.01X DPBS-10 mM EDTA targeting a concentration of 300 µM. Dibenzocyclooctyne (DBCO)-maleimide stock was prepared in anhydrous DMSO at 25 mM, added in 4-fold molar excess to the ssDNA and incubated for 2 h. In parallel, 2 mg of peptide was dissolved in Milli-Q and added in a 1:1 (*v*/*v*) ratio to Pierce™ Immobilized TCEP Disulfide Reducing Gel and incubated on a shaking platform for 90 min. Zeba™ Spin Desalting Columns 7 K were used to remove excess DBCO-maleimide according to the manufacturer’s instructions. The solution was incubated for 2 h, characterised and stored at −20 °C in solution.

### 4.3. DNA Scaffold Assembly

Bivalent, rigid DNA scaffolds were adapted from Mohri et al. [67] with sequence designs based on the desired end-to-end distances. All ssDNA sequences were submitted to NUPACK analysis to confirm correct assembly. Cartoons of the bivalent scaffolds were prepared with the Illustrate tool [68] using as input de novo models made with vHelix [69] in Maya ver. 2018b and converted to all atomic PDB through tacoxDNA [70] webserver. All bivalent scaffolds were subjected to 4–20% Native PAGE characterization, 2 h 30, 120 V. For the longer scaffolds, i.e., Bivalent-24, Bivalent-36, the core stands presenting handles were first annealed with 1.2X excess of a fluorophore-conjugated strand to ensure complete annealing of the fluorescent dyes to the Bivalent Scaffolds. Anti-handle strands that were peptide ligand conjugated prior (S4,S6) to this were then annealed in a second step. For the Bivalent-7, the core strands were directly conjugated to the peptide ligands and annealed with a 1.2X excess of the fluorophore-conjugated strand in a one-pot reaction.

### 4.4. Confocal Cell Imaging

HUVEC, CHO and HeLa cells were seeded at 3000 cells per well in 96 well ibidi angiogenesis µ-plates. Cells were serum-starved incubated overnight at 37 °C, 5% CO_2_, 95% relative humidity. The following morning, cells were incubated in full media (supplemented with activator SNAKA51 primary antibody 1 µg mL^−1^ for activation conditions) for 1 h. Buffers used for fixation, staining and imaging were adapted from Strauss et al. Cells were fixed in 2% PFA and 1X cytoskeleton buffer (1 M NaCl, 0.1 M PIPES, 30 mM MgCl_2_, 10 mM EGTA, 10 mM sucrose) for 15 min then washed with DPBS + 0.05%Tween. When non-activated, cells were incubated with SNAKA51 primary antibody for 30 min prior to the following steps. DNA scaffolds were added to cells at 7.5 µM in Bivalent Scaffold Staining Buffer and incubated for 30 min. Cells were staining with Goat Anti-Mouse IgG H&L Alexa Fluor^®^ 488 secondary antibody and DAPI prior to imaging. Cells were washed with Bivalent Scaffold Buffer and stored in Bivalent Scaffold Imaging Buffer for confocal imaging. Confocal imaging was conducted at the EPFL Bioimaging and Optics Platform on a Zeiss LSM980 with Colibri 5 illumination for fluorescence and ZEN Blue software ver. 3.4.91. The buffers used were the following: (i) Bivalent Scaffold Staining Buffer (In PBS 1X:10 mM MgCl_2_, 0.05% Tween-20, 1% BSA, 1 mM dextran sulfate, 0.2 mg/mL sheared salmon sperm DNA) (ii) Bivalent Scaffold Buffer (10 mM MgCl_2_, 5 mM TRIS, 1 mM EDTA) (iii) Bivalent Scaffold Imaging Buffer (In PBS 1X: 0.5M NaCl, 10 mM MgCl_2_, Trolox 1X, PCA 1X, PCD 1X)

### 4.5. Receptor Distribution Heatmaps

Confocal microscopy images were acquired in 3 channels, DAPI, Cy5 and Alexa488. The images were acquired as z-stacks (0.2 µm) of 15 images. Images were post-processed in FiJi software as follows in all channels for a minimum of 9 images per condition: (i) sum slices (ii) measured for signal % area and integrated signal density for plots. The heatmaps were prepared in MATLAB ver. R2018b through application of a custom colormap (reference values reported in (Supplementary S3)) after capping and normalization of the pixel’s intensities to the 98th percentile.

### 4.6. Selectivity Quantification

To quantify scaffold selectivity, we calculated the integrated density on the cell, defined with a mask based on antibody SNAKA51 presence. The quantification of the Cy5 signals was done identically for all datasets (9 per cell type and scaffold condition) using MATLAB: (i) Segmentation of cells from background using hard thresholding and morphological operators on the SNAKA51 channel, (ii) segmentation of the nuclei using the same approach in the DAPI channel, (iii) summation of the Cy5 signal in the slices when present within the defined mask of (i) but not (ii), (iv) computation of average background intensity and hard thresholding (v) computation of the average intensity of the Cy5 signal. Resulting images were exported to FiJi and identical LUT for Cy5 were applied. For statistical analysis (Supplementary S9) an ANOVA test between activated and resting conditions was applied as well as *t*-tests between each spatial scaffold in resting and activated state.

### 4.7. In-Silco Simulation and Analysis of DNA Scaffolds

Original topology and configuration files prepared with vHelix were converted in oxDNA topology and configuration files using tacoxDNA. Simulations were performed using the oxDNA simulations software with the oxDNA2 forcefield [71]. When not clearly stated, parameters for the simulations in the following pipeline were kept at their standard value and 12 replicas were performed for each structure.

At first, we performed a 1e7 steps long minimization in which the maximum force acting on the backbone was kept to 5. Then we performed 4 relaxation runs in the NVT ensemble in which we progressively warmed up the structure to 25 °C and lifted the limits on the maximum force. The respective combinations of temperature, time step and total number of steps for each relaxation run were: (i) 1 °C, 5 × 10^−4^, 5 × 10^6^, (ii) 10 °C, 5 × 10^−4^, 5 × 10^5^, (iii) 20 °C, 5 × 10^−4^, 5 × 10^5^, (iii) 20 °C, 5 × 10^−4^, 5 × 10^5^, (iv) 25 °C, 5 × 10^−4^, 5 × 10^5^. For the final production runs we used 25 °C, 1 × 10^−3^, 4.0 × 10^7^ and saved configurations for analysis every 5 × 10^3^ steps, excluding the first 0.5 × 10^7^ steps. The topologies and trajectories were then used as input for an inhouse Python script to generate files in PDB, PSF and MDCRD format for analysis in physical units using VMD [72]. Removal of periodic boundary conditions, alignment of the trajectories, calculation of the RMSF and end-to-end distances distributions were computed using inhouse scripts. As reference for the end-to-end distances, we used the center of masses of the second-last base-pair in each functionalizing arm.

## Data Availability

Not applicable.

## Supplementary Materials

The following supporting information can be downloaded at: https://www.mdpi.com/article/10.3390/molecules27154968/s1, S1: Weka analysis FiJi plugin; S2: Image processing workflow and scripts for receptor distribution analysis; S3: Table of reference values for colourmap generation; S4: all DNA strand sequences; S5: Details on Peptide synthesis and characterization (RGD and RAD) and ssDNA coupling; S6: Characterization of the DNA bivalent scaffolds; S7: Analysis of the working concentration regime for bivalent scaffold binding; S8: RAD non-binding control to confirm no a-specific interactions. S9. Statistical analysis of selectivity.

## Sample Availability

Samples of the compounds used in this study are not available from the authors.
