# Supplementary material for: Selective Integrin α5β1 Targeting through Spatially Constrained Multivalent DNA-Based Nanoparticles
Source: Molecules. 2022 Aug 4;27(15):4968. doi: 10.3390/molecules27154968 (PMC9370198; doi:10.3390/molecules27154968)

Supplementary Information for:

Selective integrin $\alpha_5\beta_1$ targeting through spatially constrained multivalent DNA-based nanoparticles

Eva E. Kurisinkal[1], Vincenzo Caroprese [1], Marianna M. Koga [1], Diana Morzy [1] and Maartje M.C. Bastings[1,2]*

1 Programmable Biomaterials Laboratory, Institute of Materials, School of Engineering, Ecole Polytechnique Fédérale Lausanne, Lausanne, 1015, Switzerland

2 Interfaculty Bioengineering Institute, School of Engineering, Ecole Polytechnique Fédérale Lausanne, Lausanne, 1015, Switzerland.

* Correspondence: maartje.bastings@epfl.ch

Table of Contents:

S1. Weka analysis FiJi plugin
S2. Image processing workflow and scripts
S3. Table of reference values for colourmap generation
S4. Table of DNA sequences for bivalent scaffolds
S5. Peptide synthesis and Characterization (RGD and RAD) and ssDNA coupling
S6. Characterization of the DNA tripods
S7. Working concentration regime for bivalent binding
S8. RAD non-binding ligand to confirm no a-specific interactions
S9. Statistical analysis of selectivity

S1. Weka analysis FiJi plugin

Images are segmented prior with the FiJi Trainable Weka Segmentation plugin, classifying foreground (cell) versus background (well). Fluorescent images and trained cell models are used as input. The script output: (i) A pixel value histogram for each image with foreground and background split. (ii) Cell probabilities from the Weka segmentation. (iii) Masks used to segment the image.

```python
#@ File(label="Input directory of brightfield", description="Select the directory with input brightfield images to use in weka.", style="directory") inputDirBFW
#@ File(label="Input directory of fluorescent", description="Select the directory with input fluorescent images to take data from.", style="directory") inputDirFluoW
#@ File(label="Output directory", description="Select the output director.y", style="directory") outputDirW
#@ File(label="Model file", description="Select the location of the .model file.", style="file") modelFileW
#@ Boolean(label="Use existing cell probabilities", description="Check if Weka model has already been applied and cell probabilites images exist.") useProb
#@ Float(label="Select model certainty", description="Threshold for the probability images to be converted into mask.", min=0, max=1) threshCertainty

from ij import IJ, ImagePlus
from ij.io import FileSaver
from ij.plugin import ImageCalculator
from ij.process import ImageConverter
from trainableSegmentation import WekaSegmentation

import os
import csv


''' FUNCTIONS '''
def save_as_tif(iplus, outDirectory):
    '''
    Saves an ImagePlus in the location specified by outDirectory
    '''
    outputFn = os.path.join(outDirectory, iplus.title + ".tif")
    FileSaver(iplus).saveAsTiff(outputFn)
    IJ.log("---Weka Analysis--- Saved image to: " + outputFn)

def to_csv(data, header, filename, outDirectory):
    '''
    Saves the speficied data into outDirectory
    '''
    #output filename
    outfn = os.path.join(outDirectory, filename)

    #write data lines in the csv
    with open(outfn, "wb") as csv_file:
        csv_writer = csv.writer(csv_file, delimiter=',')
        csv_writer.writerow(header)
        for row in data:
            csv_writer.writerow(row)

    #close and log operation success
    csv_file.close()
    IJ.log("---Weka Analysis--- Wrote csv to: " + outfn + ".")

def apply_weka(iplus):
    '''
    Applies the current weka model and saves probability images
    '''
    #remove potential DUP_ if image is duplicated (which it should to avoid unwanted modifications) and then remove the file ending
    iplus.title = imageTitle + "_cell_probability"

    #get the probabilities for each class
    iplus_classified = weka.applyClassifier(iplus, 0, True)

    #we are only interested in taking the first image in the stack (probability of being a cell), we save it in case
    iplus_prob = ImagePlus(iplus.title, iplus_classified.getImageStack().getProcessor(1))
    save_as_tif(iplus_prob, probDirString)

    return iplus_prob

def create_mask(iplus):
    '''
    Takes a cell probability image and transforms it into a binary image that can be used as a mask
    '''
    #remove potential DUP_ if image is duplicated (which it should to avoid unwanted modifications) and then remove the file ending
    iplus.title = imageTitle + "_mask"

    #threshold image with a set value which represents the probability of that pixel to be a cell
    IJ.setThreshold(iplus, threshCertainty, 1, "Black & White")
    IJ.run(iplus, "Convert to Mask", "")

    #perform binary operations to make mask better
    IJ.run(iplus, "EDM Binary Operations", "iterations=2 operation=dilate")
    IJ.run(iplus, "EDM Binary Operations", "iterations=2 operation=erode")

    #save image for later
    save_as_tif(iplus, masksDirString)

    return iplus

def analyse(iplus, mask):
    '''
    With the original cell image and the mask created with create_mask(), applies analysis to get histogram of foreground and also background.
    '''
    all_data = []

    #write header for the csv file
    header = ['Region','Area (px)']
    header.extend(['hist_'+str(i) for i in range(256)])

    #foreground
    foreground = ic.run("and create", iplus, mask)
    hist = foreground.getProcessor().getHistogram()
    hist[0] = 0 #we remove the pixel with value of 0 to get rid of the background so it doesn't hinder our data
    if gotCalibration:
        data = ["Foreground", sum(hist), sum(hist)*cal.pixelWidth*cal.pixelHeight]
    else:
        data = ["Foreground", sum(hist)]

    data.extend(hist)
    all_data.append(data)

    #background
    background = ic.run("zero create", iplus, mask)
    hist = background.getProcessor().getHistogram()
    hist[-1] = 0 #we remove the pixel with value of 255 to get rid of the foreground so it doesn't hinder our data
    if gotCalibration:
        data = ["Background", sum(hist), sum(hist)*cal.pixelWidth*cal.pixelHeight]
    else:
        data = ["Background", sum(hist)]
```

```python

    data.extend(hist)
    all_data.append(data)

    #write header for the csv file
    if gotCalibration:
        header = ['Cell number', 'Area (px)', 'Area (unit^2)'] #suddenly impossible to write µm due to encoding when it worked fine before, no other options work but to ignore it
    else:
        header = ['Cell number', 'Area (px)']

    header.extend(['hist_'+str(i) for i in range(256)])

    to_csv(all_data, header, imageTitle + ".csv", os.path.join(outputDirString, "csv"))
''' END '''

''' BEGINNING ROUTINE '''
#shorthand for weka (cuter)
weka = WekaSegmentation()

#same for calculator
ic = ImageCalculator()

#get strings of the directories, and create them if non existant
inputDirBFString = inputDirBFW.getCanonicalPath()
inputDirFluoString = inputDirFluoW.getCanonicalPath()
outputDirString = outputDirW.getCanonicalPath()
probDirString = os.path.join(outputDirString, "cell_probabilities")
csvDirString = os.path.join(outputDirString, "csv")
masksDirString = os.path.join(outputDirString, "masks")
modelFileString = modelFileW.getCanonicalPath()

for dirString in [probDirString, csvDirString, masksDirString]:
    if not os.path.exists(dirString):
        os.mkdir(dirString)
        IJ.log("---Weka Analysis--- Created output directory: " + dirString)

#load the classifier to use on the images only if probabilities don't already exist
if not useProb:
    IJ.log("---Weka Analysis--- Loading classifier")
    weka.loadClassifier(modelFileString)
    IJ.log("---Weka Analysis--- Classifier loaded")

gotCalibration = False
''' END '''

''' BATCH LOOP '''
#loop around each image and apply the classification, with counter
c = 0
for imageFn in os.listdir(inputDirBFString):
    if imageFn.endswith(".tif") or imageFn.endswith(".tiff") or imageFn.endswith(".jpg") or imageFn.endswith(".jpeg") or imageFn.endswith(".png"):
        imageBF = IJ.openImage(os.path.join(inputDirBFString, imageFn))
        imageTitle = imageBF.title.rsplit(".",1)[0] #title with file extension to use throughout the code
        IJ.log("---Weka Analysis--- Processing " + imageTitle)

        #get scaling to return values and perform analysis in units, prioritises global scale over local one (just like Fiji)
        cal = imageBF.getCalibration()
        if cal.unit=="pixel":
            gotCalibration = False
            IJ.log("---Weka Analysis--- Did not find a scale. Consider setting a global or local scale if you want the data in something else than pixels.")
        else:
            gotCalibration = True
            IJ.log("---Weka Analysis--- Found a scale, using that for calculations and particle analysis.")

        #open fluorescent image and throw error if cannot find the fluorescent image
        imageFluo = IJ.openImage(os.path.join(inputDirFluoString, imageFn))

        if imageFluo is None:
            IJ.log("---Weka Analysis--- Could not find fluorescent image for " + imageFn + ". Please make sure that it has the same name as the brightfield image.")
            continue
        else:
            #convert to 8bit to be sure that values are in normal range
            ImageConverter(imageFluo).convertToGray8()

        c += 1
        #if cell probabilites already exist, use those instead of apply Weka which can take a long time
        if useProb:
            cellProbImage = IJ.openImage(os.path.join(probDirString, imageTitle + "_cell_probability.tif"))
        else:
            cellProbImage = apply_weka(imageBF.duplicate())

        mask = create_mask(cellProbImage)
        analyse(imageFluo.duplicate(), mask)

IJ.log("---Weka Analysis--- Found and processed " + str(c) + " images")
''' END '''
```

S2. Image processing workflow and scripts

For the modelling of receptor distributions, 3 images were selected at random per condition and subjected to the workflow detailed below in Wolfram Mathematica. We assumed the presence of two normal distributions of pixel intensities corresponding to background and signal respectively and proceed with relative fitting. Pixel intensity values lower than that of the intersection of the distributions are set to zero (background). The intensities of the pixels in the image are summed. Then, the intensity value attributed per receptor is calculated by dividing the total pixel intensity of an image by the corrected number of integrin $\alpha_5\beta_1$ receptors. Next, the integrin $\alpha_5\beta_1$ receptors are distributed by individual pixel intensity and the inter-receptor distances within a pixel (3.115 pixels µm-1) are calculated assuming an even distribution.

First set the directory to whatever working directory you want. NotebookDirectory[] will be the place where the notebook is saved.

```
In[ ]:= SetDirectory[NotebookDirectory[]]
Out[ ]=

In[ ]:=
Out[ ]=
```

Set the name of the file to perform calculations on.

```
dataFn  = "Data_FilePath";
imageFn = "Image_FilePath";
```

Pre calculations

These new lines import/calculate import values that are used throughout the notebook.

Scale

The pixel length is the length of one side of a pixel. This is only needed for the receptor spacing calculations.

```
In[]:= pixelLength = 320 nm
```

Starting index

Because the data can contain two areas if it came with units, we calculate the starting index of the histogram data.

```
In[]:= header = Import[dataFn][[1]];
       strtIdx = Position[header, "hist_0"][[1, 1]]
Out[]=
```

Background value

First, we import the data from Fiji where the histograms for background and foreground are kept. We take the background histogram.

```
In[]:= rawHistData = Import[dataFn][[2 ;;]];
       backroundHistData = rawHistData[[2, strtIdx ;;]];
```

Since backroundHistData is a histogram we multiply each vale by their occurrence leading us to allBackgroundValues. We use values from 0 to 255 here because it should coincide with the integrated density calculation found below.

```
In[]:= allBackgroundValues =
         Flatten[MapThread[ConstantArray, {Range[0, 255], backroundHistData}]];
```

We can then take the mean of this to get the average background value. This calue will be between 0 and 255.

```
In[]:= backgroundValue = N[Mean[allBackgroundValues]]
Out[]=
```

Receptors per pixel value

The value totalReceptors is taken directly from prior calculations. Integrated density will sum all the pixel values for each cell, subtracting the background value (calculated above) with it. When we subtract the background value, if pixels in the cells are underneath this level, we just put it to 0. pixelValues is just a flattened list of all the pixel values that are in the cell calculated from the histogram in the csv file.
It is important that the totalReceptor number is calculated from values of 0 to 255 as this is how the integrated density is calculated.

```
totalReceptors =
```

```
In[]:= foregroundHistData = rawHistData[[1, strtIdx ;;]];
       pixelValues = Flatten[MapThread[ConstantArray, {Range[0, 255], foregroundHistData}]];

In[]:= integratedDensity = N[Total[If[# < 0, 0, #] & /@ (pixelValues - backgroundValue)]]
Out[]=

In[]:= valuePerReceptor = integratedDensity / totalReceptors
Out[]=
```

Inter-receptor spacing histogram

First, let's define a function, getSpacingHistograms[], which will calculate, for each pixel, the number of receptors that is should have in it. Further explanation is found inside the function.

```
In[∘]:= getSpacingHistograms[cellPixelsValues_, pixelArea_, fractionOccupied_] :=
  Block[{receptorsPerPixel, areaPerReceptor, spacingBetweenReceptors},
   (*first, for every pixel value, we remove the background,
   divide it by our valuePerReceptor and round it to get the number of
    receptors in each pixel, if the value is below 0 we set it to 0*)
   receptorsPerPixel = If[Round[#] ≤ 0, Nothing, Round[#]] & /@
     ((cellPixelsValues - backgroundValue) / valuePerReceptor);
   (*taking into account the fraction of the area of each pixel that a
    receptor can occupy, we calcualte the areas for each receptors*)
   areaPerReceptor = (fractionOccupied pixelArea / #) & /@ receptorsPerPixel;
   (*we can then, assuming a circular occupation,
   calculate the distance for each receptor*)
   spacingBetweenReceptors = Sqrt[4 * areaPerReceptor / Pi];
   (*as is done further up in the notebook,
   we translate histogram values to a list of the occurences and return this*)
   Flatten[MapThread[ConstantArray, {spacingBetweenReceptors, receptorsPerPixel}]]
  ]
```

We calculate the fraction of area of a pixel accessible to a receptor, factoring a jamming limit of 0.54. The calculated values of each pixel is plotted in a histogram.

```
In[∘]:= pixelArea = pixelLength^2;
fractionOccupied = .54;
spacingHists = getSpacingHistograms[pixelValues, pixelArea, fractionOccupied];
meanSpacingHists = Mean[spacingHists];
```

To check if the results are correct, totalNumber and the length of spacingHists should be roughly similar.

```
In[∘]:= totalReceptors
Length[spacingHists]
```

```
In[∘]:= receptorHist = Histogram[spacingHists, 40,
   "Probability", PlotRange → {{0, 60}, Automatic}, FrameLabel →
    {Style["Receptor spacing (nm)", Black, 15], Style["Frequency", Black, 15]},
   Frame → True, GridLines → Automatic, ImageSize → Large,
   PlotLabel → Style["Histogram of receptor spacing\nMean: " <>
      ToString[meanSpacingHists] <> "nm", Black, 15]]
```

Out[∘]=

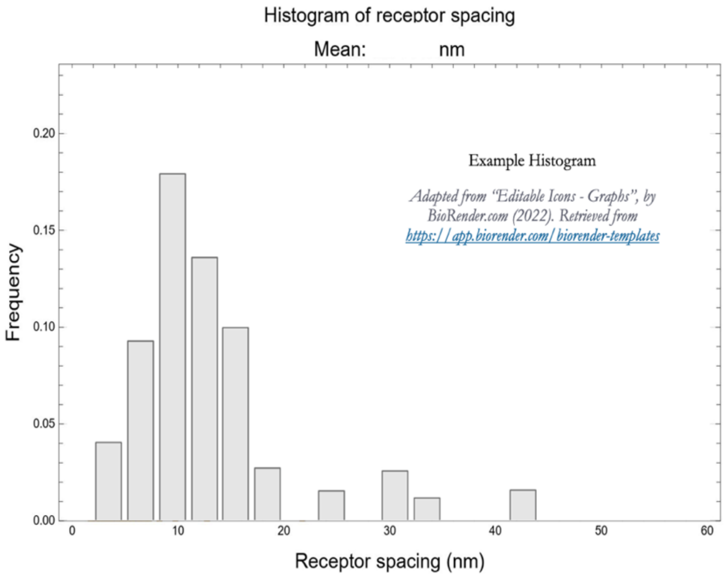

Save the histogram wherever needed.

```
In[∘]:= Export["visualisations/receptor_histograms.png", receptorHist, ImageResolution → 500]
Out[∘]= visualisations/receptor_histograms.png
```

If you want to make histograms in another program, evaluate the next cell to save the histogram data into a csv file.

```
In[ ]:= binWidth = 10;
binStart = Min[spacingHists];
binEnd = Max[spacingHists] + binWidth;

In[ ]:= histList = HistogramList[spacingHists, {binStart, binEnd, binWidth}]

Out[ ]=

In[ ]:= Export["visualisations/histogram_data.csv", histList]

Out[ ]= visualisations/histogram_data.csv
```

S3. Table of reference values for colourmap generation

Listed below are the reference values used to generate our custom colormap through a script replicating the approach of the function Blend in Mathematica.

Starting point	R	G	B	Ending point	R	G	B
0.00000	0.18824	0.12549	0.51373	0.15294	0.27490	0.18230	0.72730
0.15294	0.40060	0.30210	1.00000	0.32157	0.80060	0.74900	0.99960
0.32157	0.10460	0.70040	1.00000	0.49020	0.65277	0.93243	1.00000
0.49020	0.20565	0.96565	0.08898	0.66275	0.72212	1.00000	0.55815
0.66275	0.97227	0.93823	0.19231	0.83137	1.00000	0.97672	0.59822
0.83137	0.90980	0.40784	0.40784	1.00000	0.95686	0.72157	0.72157

S4. Table of DNA sequences for bivalent scaffolds

All ssDNA strands listed below were ordered from Integrated DNA Technologies, Coralville, Iowa, USA, with modifications when indicated for peptide conjugation.

Sequence Name	Sequence	Modification
Bivalent-7-1	GTTGCTAGTGGTGTCCAAAC	5′ azide
Bivalent-7-2	GTTTGGACACTCAGCCTAACGCTAG	5′ azide
Bivalent-7-3	CCATAGACTAGCAACTTTCACCCTAGCGTTAGGCTGACACTAGCAAC	
Bivalent-24-1	TTCCTCTACCACCTACATCACCTAGCGTTGCTAGTGGTGTCCAAACGCTAG	
Bivalent-24-2	TTCCTCTACCACCTACATCACCTAGCGTTTGGACACTCAGCCTAACGCTAG	
Bivalent-24-3	CCATAGACTAGCAACTTTCACCCTAGCGTTAGGCTGACACTAGCAACGCTAG	
Bivalent-36-1	TTCCTCTACCACCTACATCACGCGTTGCTAGTGTCAGCCTAGCGTTGCTAGTGGTGTCCAAACGCTAGAATACTGCAGTACGATC	
Bivalent-36-2	TTCCTCTACCACCTACATCACGATCGTACTGCAGTATTCTAGCGTTTGGACACTCAGCCTAACGCTAG	
Bivalent-36-3	CCATAGACTAGCAACTTTCACCCTAGCGTTAGGCTGACACTAGCAACGCTAGGCTGACACTAGCAACGC	
Peptide antihandle	GTGATGTAGGTGGTAGAGGAA	3′azide
Dye antihandle	GGTGAAAGTTGCTAGTCTATGG	3′ Cy5

S5. Peptide synthesis and Characterization (RGD and RAD) and ssDNA coupling

65µmol Fmoc-based solid phase peptide synthesis (SPPS) was conducted of the following peptides (GRGDSGGGC, GRADSGGGC) on Fmoc-Rink Amide MBHA resin. The entire synthesis was conducted in solid phase synthesis vessels under a steady stream of Argon. Fmoc deprotection was performed by mixing the resin in a 20% (v/v) NMP supplemented with 0.1M HOBt for five minutes. This was followed by six washing steps of NMP. For each amino acid coupling, four molar equivalents relative to resin loading of Fmoc-protected amino acid, four molar equivalents of HBTU in NMP and 16 molar equivalents of DIPEA in NMP were added to the reaction chamber and mixed at room temperature. The first 5 amino acid couplings were conducted for 1 hour and the remaining for 2 hours. The efficiency of coupling and deprotection was assessed as previously reported.18 After the last deprotection and washing step, an additional washing step with 4 times 8mL of DCM was conducted. The resin was then dried under compressed air for 30 minutes. Peptide cleavage from the resin and removal of side chain protecting groups was carried out with the following cleavage cocktail for four hours. The 2mL cleavage cocktail was composed of 90% (v/v) TFA, 2.5% (v/v) Milli-Q, 2.5% (v/v) thioanisole, 2.5% (w/v) phenol, and 2.5% (v/v) EDT. Precipitation was conducted with cold diethyl ether, and incubated for 30 minutes at -20°C. The solution and precipitate was then centrifuged at 2000 x g for 20 minutes. The precipitate was washed with 40mL cold diethyl ether and the centrifugation step repeated. Then, the precipitate was dissolved in ddH2O, lyophilised and stored at -80°C in powder form. For CF488-peptide conjugation, peptide dissolved in Milli-Q was first added in a 1:1 (v/v) ratio to Pierce™ Immobilized TCEP Disulfide Reducing Gel and incubated on a shaking platform for 90mins. To separate the TCEP Gel from the reduced peptide sample, the mix was added to Pierce™ Spin Cups and centrifuged for 1minute at 1500 x g into microcentrifuge tubes containing the CF488-maleimide, in 1.2X molar excess.

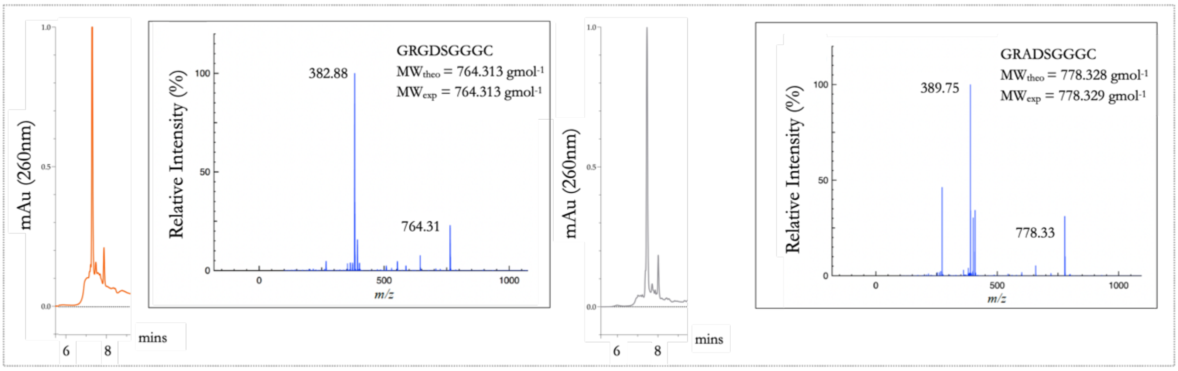

Figure S5a. HPLC chromatograms and ESI-MS of RGD(left) and RAD(right) peptides. Peptide molecular weight was analysed by ESI-MS. m/z Calcd. [M+H]+: 764.29Da Obsd. [M+H]+: 764.31Da [M+2H]2+: 382.66Da (CGGGGRGDS) m/z Calcd. [M+H]+: 778.30Da Obsd. [M+H]+: 778.33Da [M+2H]2+: 389.67Da (CGGGGRADS). The peptides synthesised were characterised by RP-HPLC. A gradient of 15% to 75%B over 24 minutes was used with A: Milli-Q with 0.1% (v/v) TFA and B: acetonitrile with 0.1% (v/v) TFA.

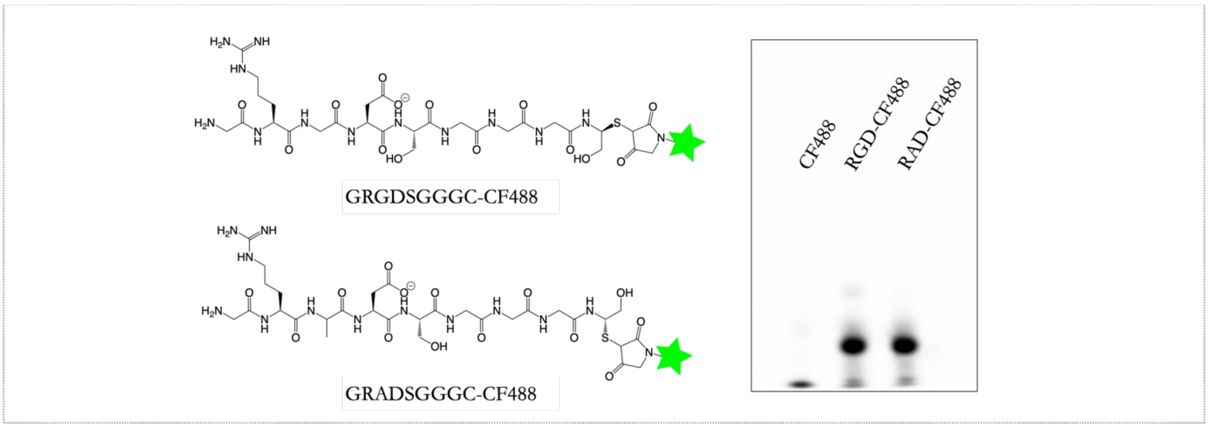

Figure S5b. Chemical structure of RGD and RAD peptides conjugated to CF488 dye. Peptide-CF488 conjugates were characterised on 4-20% Native Gels for 1h20, 120V.

S6. Characterization of the DNA tripods

Peptides were conjugated to ssDNA strands with azide modification (Table S4) for annealing to the DNA Scaffolds. All the following steps were conducted at room temperature. ssDNA was resuspended in 0.01XDPBS-10mM EDTA targeting a concentration of 300μM. Dibenzocyclooctyne (DBCO)-maleimide stock was prepared in anhydrous DMSO at 25mM, added in 4-fold molar excess to the ssDNA and incubated for 2h. In parallel, 2mg of peptide was dissolved in Milli-Q and added in a 1:1 (v/v) ratio to Pierce™ Immobilized TCEP Disulfide Reducing Gel and incubated on a shaking platform for 90mins. After incubation, Zeba™ Spin Desalting Columns 7K were used to remove excess DBCO-maleimide according to the manufacturer's instructions.3 To separate the TCEP Gel from the reduced peptide sample, the mix was added to Pierce™ Spin Cups and centrifuged for 1minute at 1500 x g into microcentrifuge tubes containing the purified ssDNA-maleimide sample. The solution was incubated for 2h, characterised and stored at -20°C in solution (**Figure S6a**).

All bivalent scaffolds were annealed in for 1h in a thermal cycler at either a one-pot (Bivalent-7) or two-pot (Bivalent-24, Bivalent-36) in Bivalent Scaffold Buffer (10mM MgCl2, 5mM TRIS, 1mM EDTA). Annealing protocol: i) Denaturation, 95°C, 2min. ii) Cooling at 65°C, 5 min. iii) Hold, 60°C, 2 min. iv) Ramp, 60°C to 20°C at 1°C/min (repeated 40X). Stored at 4°C. In the second-pot peptide-antihandles were added 1.2X in excess per handle and annealed at 37°C for 1h. The peptide liganded bivalent scaffolds were subjected to 4-20% Native PAGE characterisation (**Figure S6b**), 2h30, 120V. In Red (Cy5-Bivalent Scaffold) and blue (SYBR Gold-DNA).

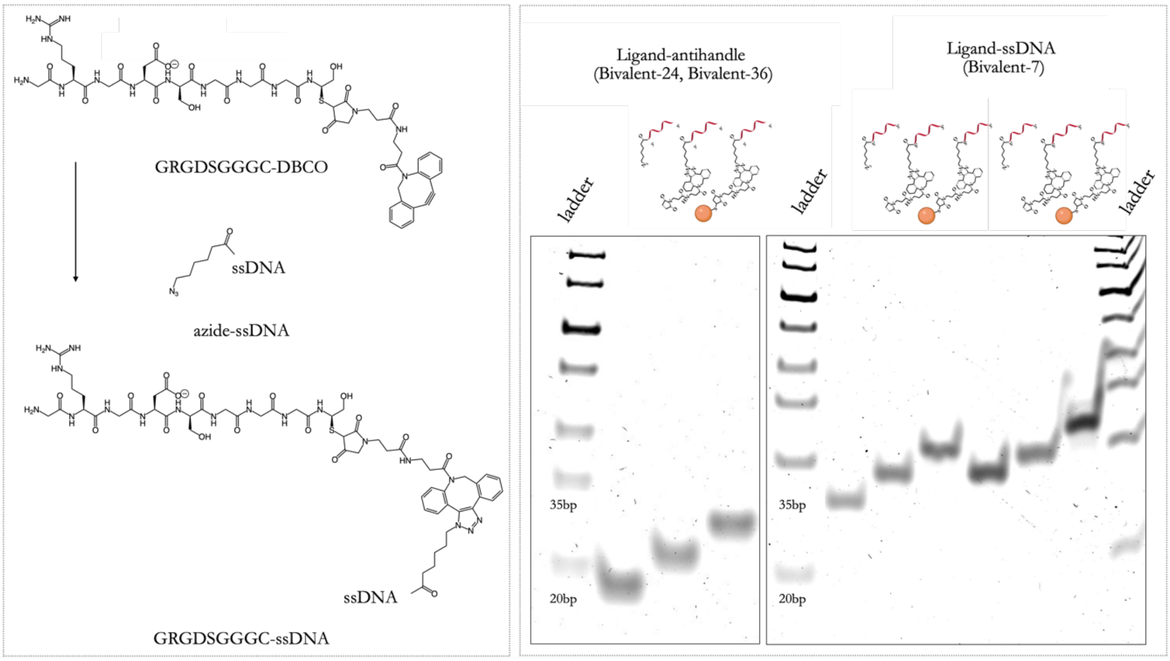

Figure S6a. (Right) SPAAC conjugation of azide modified ssDNA to cysteine bearing peptides. (Left) 20% Native PAGE gel of peptide-ssDNA conjugates. Peptides ligand functionalised ssDNA antihandles were characterized by 20% Native PAGE. 2µM, 5µL samples were loaded on 20% Native PAGE gels and run for 2h30-3h30 hours at 150V.

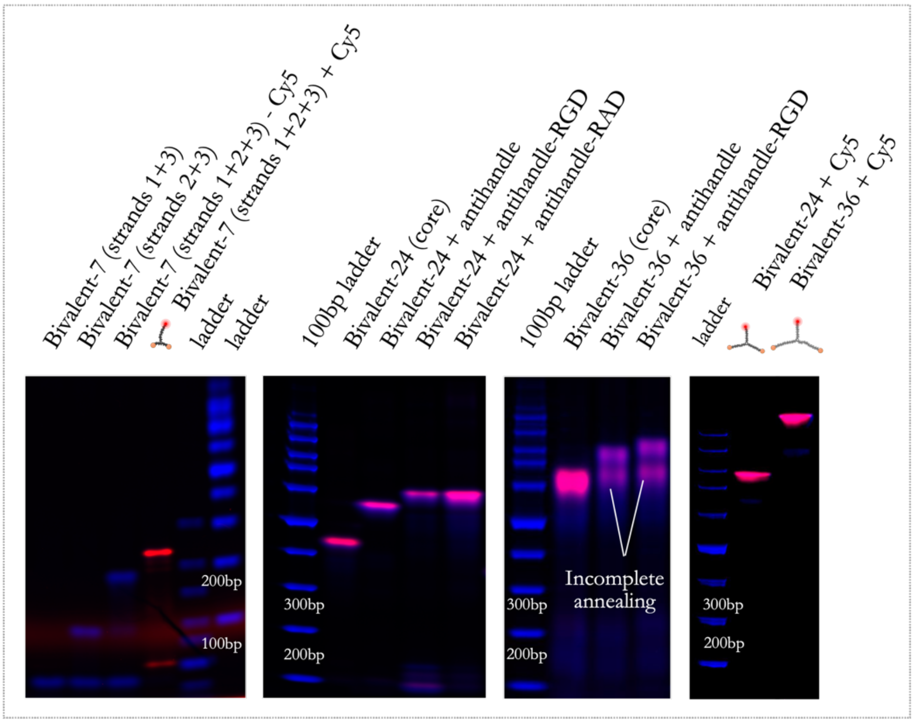

Figure S6b. Native PAGE gel characterisation of Bivalent Scaffolds.

S7. Working concentration regime for bivalent binding

HUVEC, CHO and HeLa cells were seeded in 96 well ibidi angiogenesis μ-plate at 3'000 cells/well and incubated overnight at 37°C, 5% CO_2, 95% relative humidity. The following day, cells were incubated in full media (supplemented with activator SNAKA51 primary antibody 1μg mL^{-1}) for 1h. The following steps were conducted at room temperature. Cells were fixed in 2% PFA for 15 mins. Cells were then washed with DPBS, blocked in BSA3% or BlockAid for 30 mins, Image-iT™ FX Signal Enhancer for 15 mins and DAPI (1:1000), 3 mins. Bivalent-24-RGD (**Figure S7a**) or CF488-RGD (**Figure S7b**) were added in different concentrations to the cells and incubated for 1h. The Bivalent Scaffolds were incubated in Bivalent Scaffold buffer prior to plate reader imaging. Imaging conditions: LED intensity :10; Integration time : 600ms; Camera Gain : 10. LUT Applied, Cy5 : Red; DAPI : Blue. Monovalent LUT Applied, CF488 : Green; DAPI : Blue.

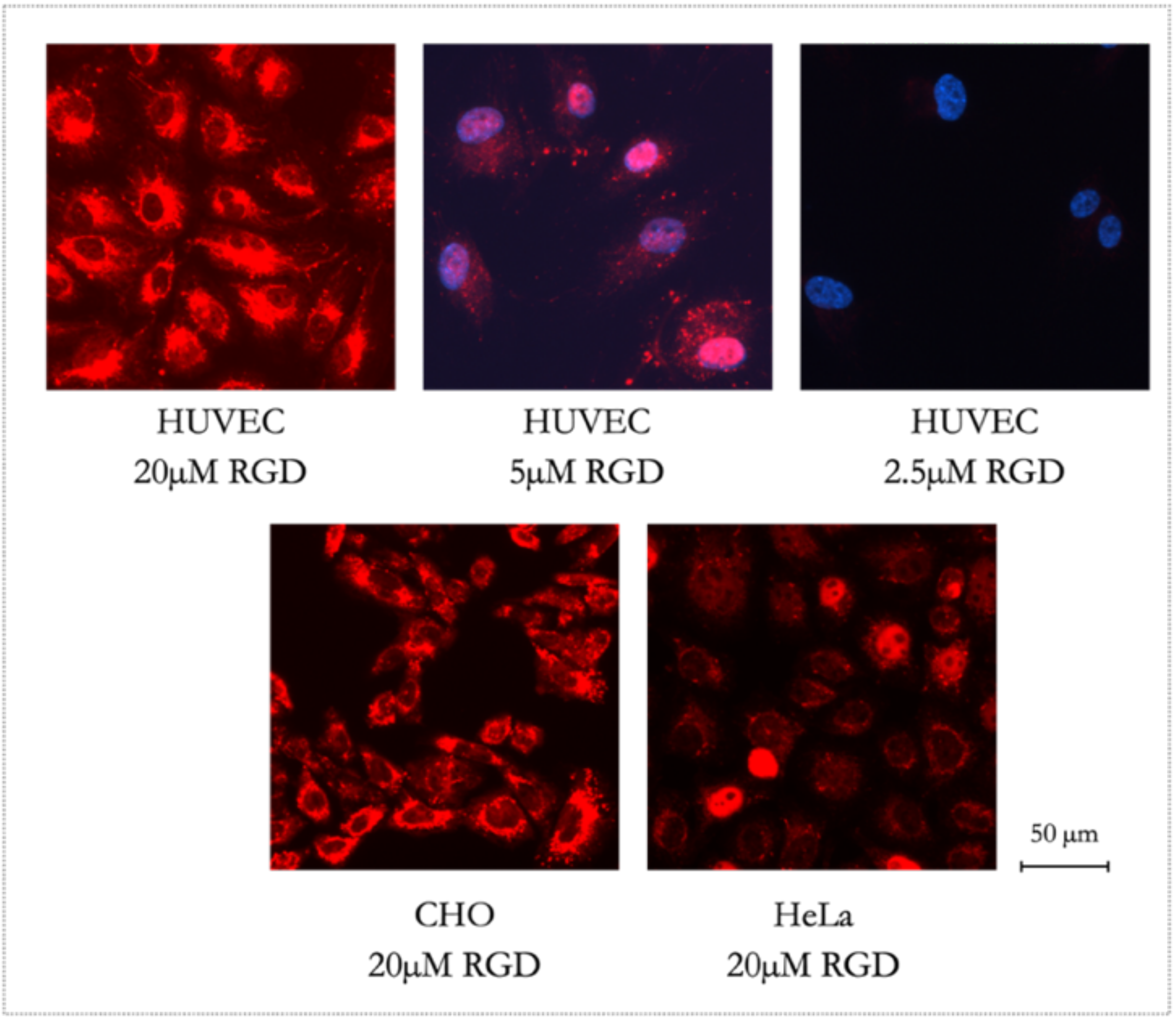

Figure S7a. (Top) Bivalent Scaffold binding concentration range on HUVECs. (Bottom) CHO and HeLa controls at 20μM Bivalent-24.

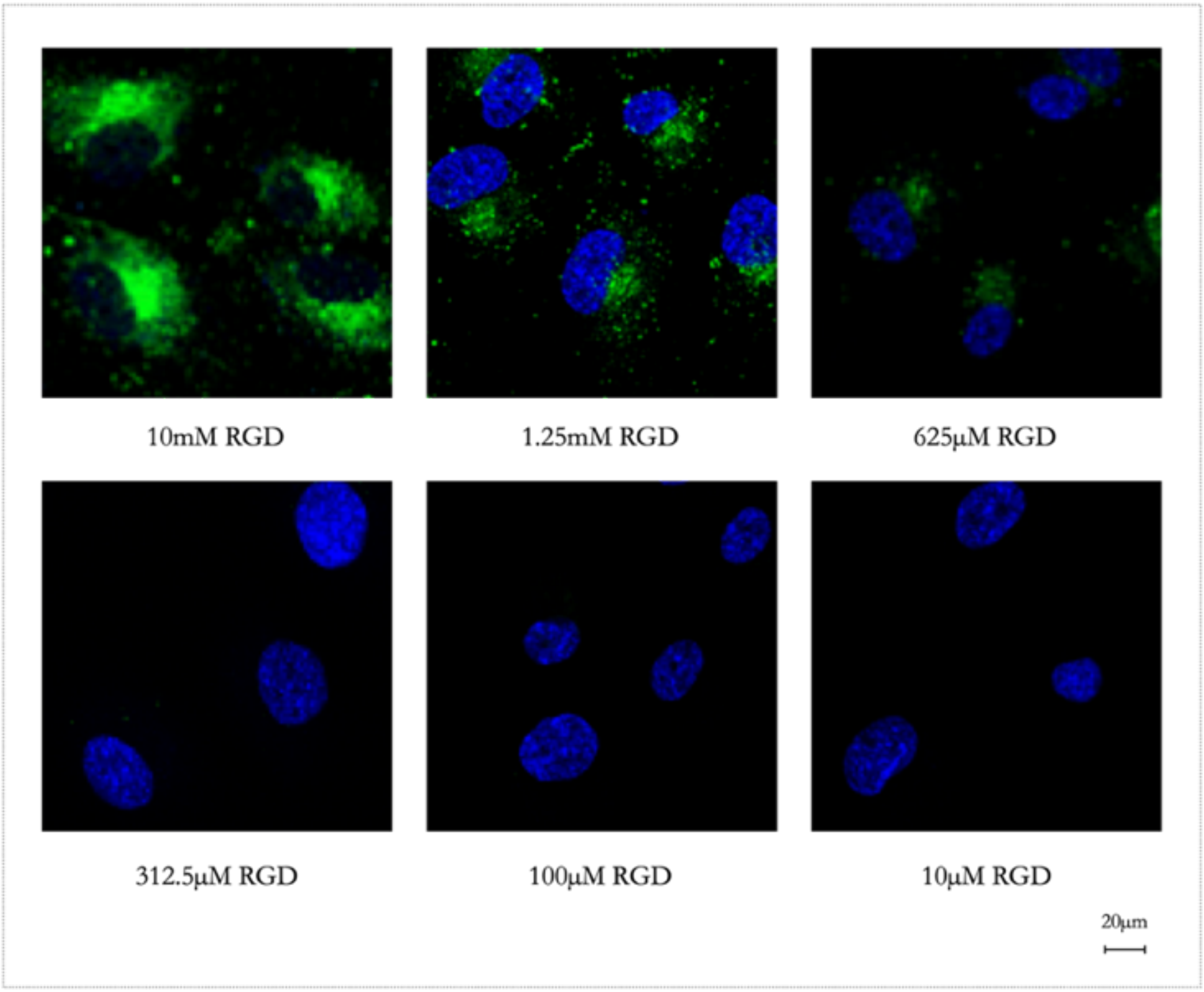

Figure S7b. Monovalent RGD binding concentration regime.

S8. RAD non-binding ligand to confirm no a-specific interactions

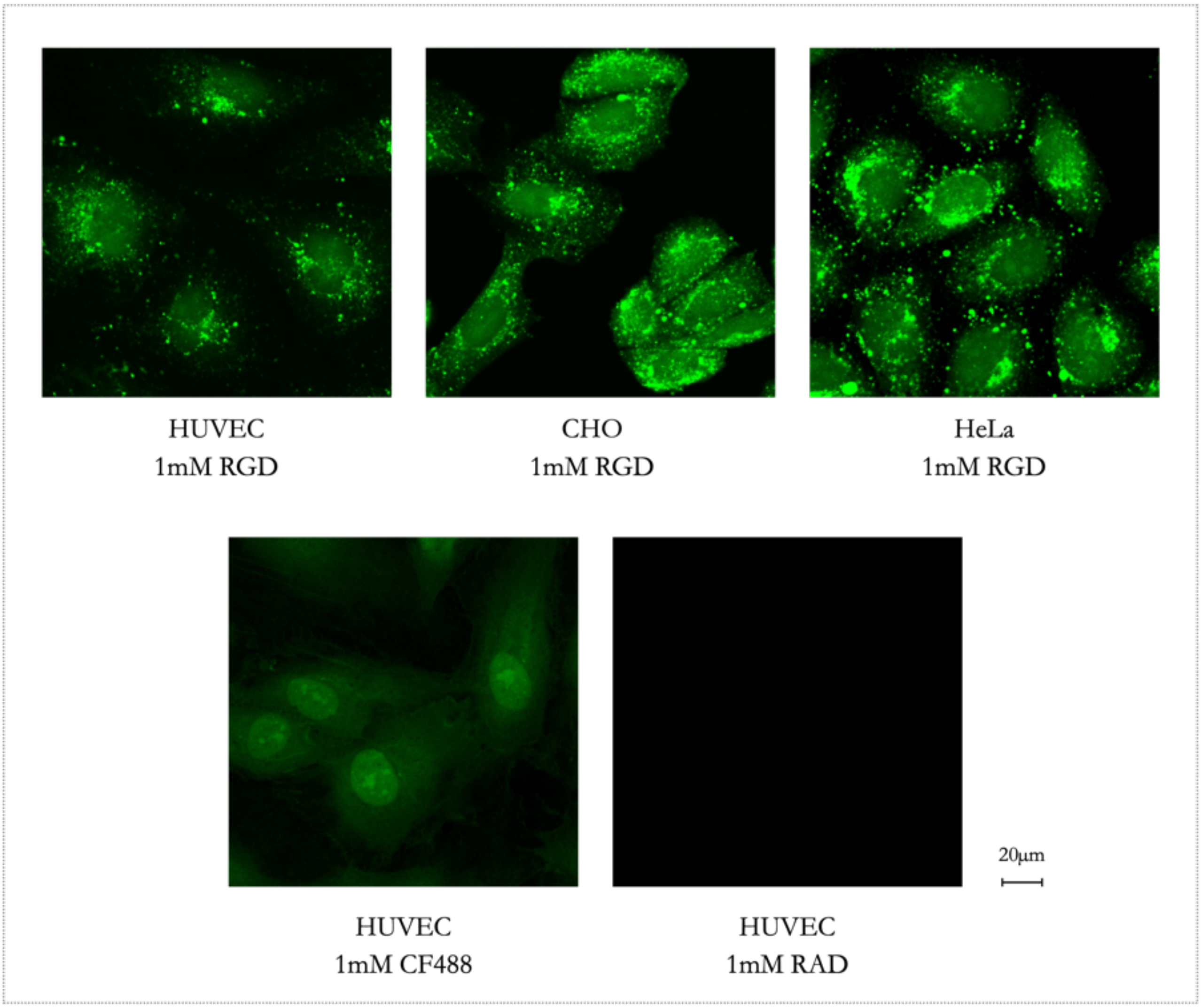

Figure S8. (Top) 1mM RGD-CF488 controls on HUVEC, CHO and HeLa. (Bottom) Free CF488 dye and RAD non-binding peptide controls. HUVEC, CHO and HeLa cells were seeded in 96 well ibidi angiogenesis µ-plate at 3’000 cells/well and incubated overnight at 37°C, 5% CO_2, 95% relative humidity. The following day, cells were incubated in full media (supplemented with activator SNAKA51 primary antibody 1µg mL^{-1}) for 1h. The following steps were conducted at room temperature. Cells were fixed in 2% PFA for 15 mins. Cells were then washed with DPBS, blocked in BlockAid for 30 mins and Image-iT™ FX Signal Enhancer for 15 mins. CF488-RGD, CF488-RAD or free CF488 dye was added at 1mM to the cells and incubated for 1h prior to confocal imaging.

S9. Statistical analysis of selectivity

Grouped 2way ANOVA between “resting conditions” and “activated state”

HUVEC:

Source of Variation	% of total variation	P value	P value summary	Significant?
Column Factor	55.31	<0.0001	****	Yes

CHO

Source of Variation	% of total variation	P value	P value summary	Significant?
Column Factor	47.55	<0.0001	****	Yes

HeLa

Source of Variation	% of total variation	P value	P value summary	Significant?
Column Factor	1.217	0.5045	ns	No

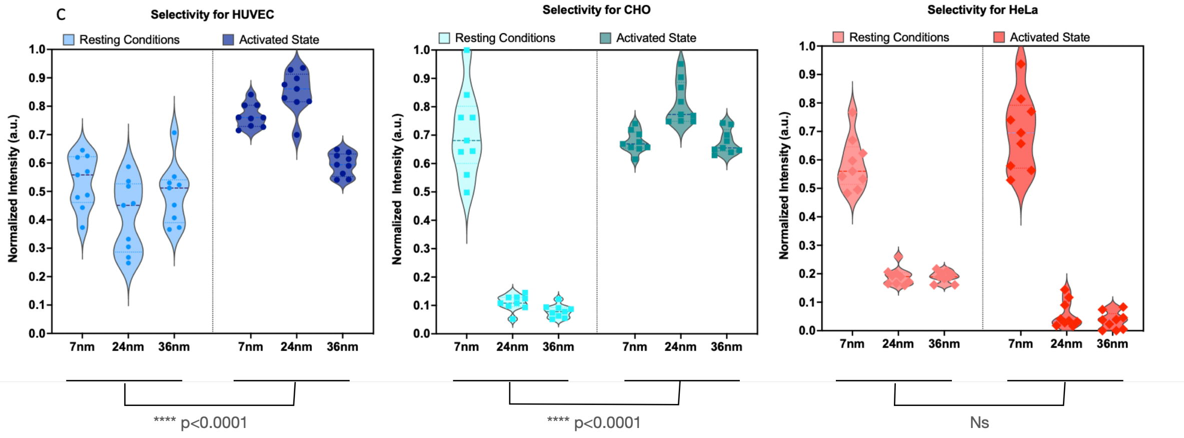

HUVEC t-test between each scaffold in resting versus activated state

Column B	7nm activated
vs.	vs.
Column A	7nm resting
Unpaired t test	
P value	<0.0001
P value summary	****
Significantly different (P < 0.05)?	Yes

Column D	24nm act
vs.	vs.
Column C	24nm rest
Unpaired t test	
P value	<0.0001
P value summary	****
Significantly different (P < 0.05)?	Yes

Column F	36nm act
vs.	vs.
Column E	36nm rest
Unpaired t test	
P value	0.0149
P value summary	*
Significantly different (P < 0.05)?	Yes

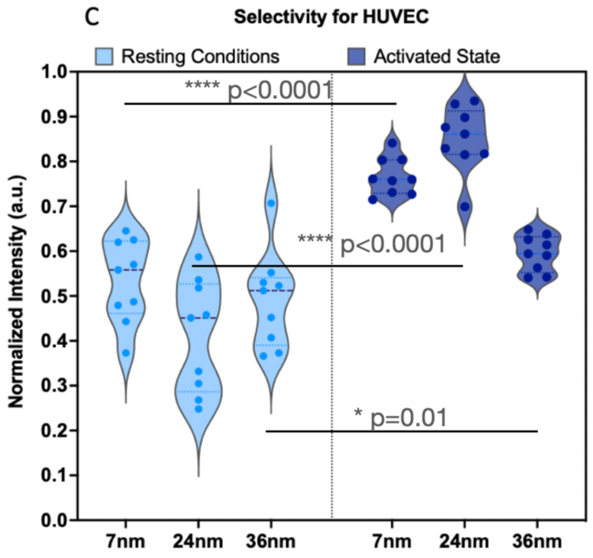

CHO t-test between each scaffold in resting versus activated state

Column B	7nm act
vs.	vs.
Column A	7nm rest
Unpaired t test	
P value	0.5308
P value summary	ns
Significantly different (P < 0.05)?	No
Column D	24nm act
vs.	vs.
Column C	24nm rest
Unpaired t test	
P value	<0.0001
P value summary	****
Significantly different (P < 0.05)?	Yes
Column F	36nm act
vs.	vs.
Column E	36nm rest
Unpaired t test	
P value	<0.0001
P value summary	****
Significantly different (P < 0.05)?	Yes

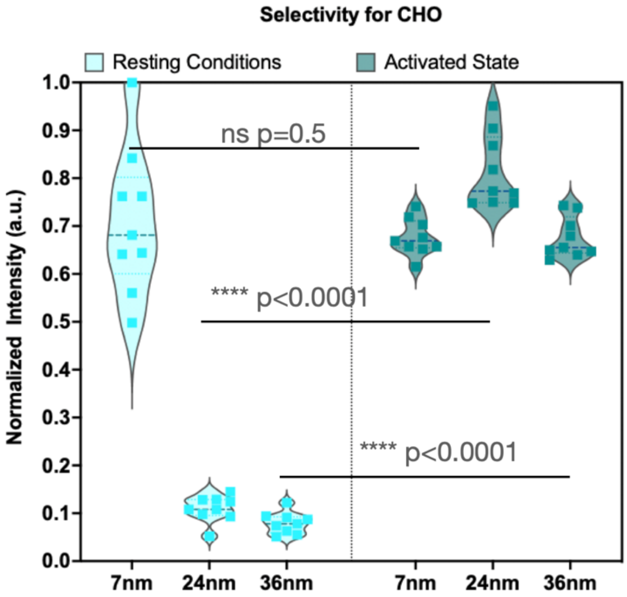

HeLa t-test between each scaffold in resting versus activated state

Column B	7nm act
vs.	vs.
Column A	7nm rest
Unpaired t test	
P value	0.0509
P value summary	ns
Significantly different (P < 0.05)?	No
Column D	24nm act
vs.	vs.
Column C	24nm rest
Unpaired t test	
P value	<0.0001
P value summary	****
Significantly different (P < 0.05)?	Yes
Column F	36nm act
vs.	vs.
Column E	36nm rest
Unpaired t test	
P value	<0.0001
P value summary	****
Significantly different (P < 0.05)?	Yes

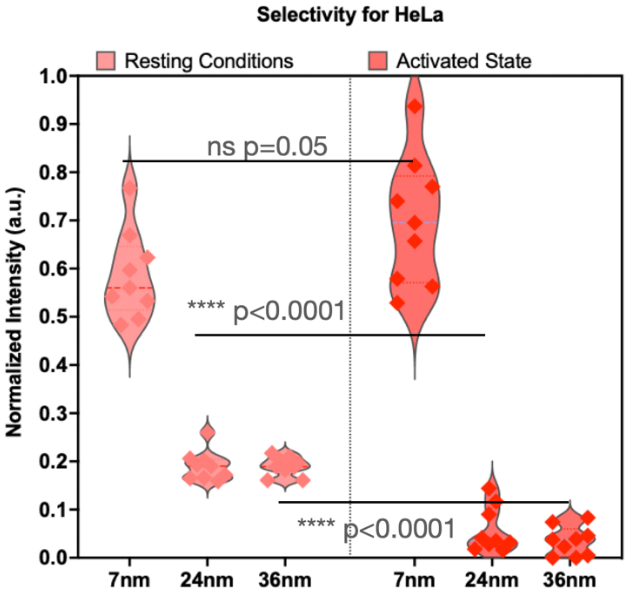

Supplement: Supplementary file 1 [file molecules-27-04968-s001.zip › molecules-1820434-supplementary.pdf]
